# Cortical structural differences following repeated ayahuasca use hold molecular signatures

**DOI:** 10.3389/fnins.2023.1217079

**Published:** 2023-10-05

**Authors:** Pablo Mallaroni, Natasha L. Mason, Lilian Kloft, Johannes T. Reckweg, Kim van Oorsouw, Johannes G. Ramaekers

**Affiliations:** ^1^Department of Neuropsychology and Psychopharmacology, Faculty of Psychology and Neuroscience, Maastricht University, Maastricht, Netherlands; ^2^Department of Forensic Psychology, Faculty of Psychology and Neuroscience, Maastricht University, Maastricht, Netherlands

**Keywords:** ayahuasca, psychedelics, 5-HT2A, transcriptomics, morphometry, ultra-high field MRI

## Abstract

**Introduction:**

Serotonergic psychedelics such as ayahuasca are reported to promote both structural and functional neural plasticity via partial 5-HT_2A_ agonism. However, little is known about how these molecular mechanisms may extend to repeated psychedelic administration in humans, let alone neuroanatomy. While early evidence suggests localised changes to cortical thickness in long-term ayahuasca users, it is unknown how such findings may be reflected by large-scale anatomical brain networks comprising cytoarchitecturally complex regions.

**Methods:**

Here, we examined the relationship between cortical gene expression markers of psychedelic action and brain morphometric change following repeated ayahuasca usage, using high-field 7 Tesla neuroimaging data derived from 24 members of an ayahuasca-using church (Santo Daime) and case-matched controls.

**Results:**

Using a morphometric similarity network (MSN) analysis, repeated ayahuasca use was associated with a spatially distributed cortical patterning of both structural differentiation in sensorimotor areas and de-differentiation in transmodal areas. Cortical MSN remodelling was found to be spatially correlated with dysregulation of 5-HT_2A_ gene expression as well as a broader set of genes encoding target receptors pertinent to ayahuasca’s effects. Furthermore, these associations were similarly interrelated with altered gene expression of specific transcriptional factors and immediate early genes previously identified in preclinical assays as relevant to psychedelic-induced neuroplasticity.

**Conclusion:**

Taken together, these findings provide preliminary evidence that the molecular mechanisms of psychedelic action may scale up to a macroscale level of brain organisation *in vivo*. Closer attention to the role of cortical transcriptomics in structural-functional coupling may help account for the behavioural differences observed in experienced psychedelic users.

## Introduction

In recent years, classical psychedelic compounds such as psilocybin, lysergic acid diethylamide (LSD) and N,N-dimethyltryptamine (DMT) have demonstrated significant utility for the treatment of neuropsychiatric disorders, including depression, anxiety, and substance-use disorders (Bogenschutz et al., 2022; D’Souza et al., 2022; Holze et al., 2023). A promise of their therapeutic efficacy is their capacity to elicit sustained behavioural and cognitive change following a single administration, making them a rapid-acting and durable treatment option (Knudsen, 2023).

Current data on classical psychedelics strongly suggest that activation of the serotonergic 5-HT_2A_ receptor not only mediates the acute hallucinogenic effects of psychedelics but also potentiates neuroplastic adaptations proposed to underlie persisting symptom improvements (Kwan et al., 2022; Vargas et al., 2023). A general umbrella term that refers to the brain’s ability to modify, change, and adapt throughout life and in response to experience, neuroplasticity arises at both functional and structural axes of organisation (Mateos-Aparicio and Rodríguez-Moreno, 2019). Ample preclinical evidence has highlighted the induction of both structural and functional plasticity in cortical neurons following the application of 5-HT_2A_ agonists and subsequent glutaminergic drive. These changes span from the promotion of immediate early genes (IEGs) such as ARC and cFOS, implicated in long-term cellular responses to external stimuli and spiking activity, to more downstream evidence of augmented synaptogenesis, neurogenesis and dendritogenesis (Calder and Hasler, 2023). In humans, these “psychoplastogenic” properties (Olson, 2018) are hypothesised to underlie neuroimaging findings in both clinical and neurotypical populaces of enduring alterations to the topography of large-scale brain functional networks following administration of a psychedelic compound (Sampedro et al., 2017; Barrett et al., 2020; Pasquini et al., 2020; McCulloch et al., 2022). For example, resting-state analyses have highlighted that 5-HT_2A_-rich higher-order functional networks exhibit greater functional interconnectedness and neural flexibility after psilocybin treatment, detectable for at least 1 week after a single dose exposure (Doss et al., 2021; Daws et al., 2022).

However, little is known regarding the impact of repeated exposure to a psychoplastogen, an important question given that (recreational) use of a psychedelic is rarely limited to a single occurences (Glynos et al., 2022). Furthermore, chronic use of a host of ultimately glutaminergic substances such as 3,4-methylenedioxymethamphetamine (MDMA), ketamine or cannabis has been frequently suggested to elicit gross alterations to brain structure (Liao et al., 2011; Lanteri et al., 2014; Müller et al., 2019; Manza et al., 2020; Robinson et al., 2023). A cultural phenomenon pertinent to the study of repeated psychedelic use is the ritualistic intake of ayahuasca by syncretic religions such as Santo Daime. Members of Santo Daime drink ayahuasca (or “Daime”) on a near-weekly basis as a religious sacrament, with membership often maintained for life (Moreira and MacRae, 2011; Labate, 2012; Hartogsohn, 2021). A psychedelic brew made from *Psychotria viridis* leaves and *Banisteriopsis caapi* vines, respectively containing the 5-HT_2A_ agonist DMT and monoamine oxidase inhibiting (MAOI) β-carboline alkaloids such as harmine, harmaline, and tetrahydroharmine (Riba et al., 2003), ayahuasca has been previously shown to promote neuroplasticity and neurogenesis, as well as elicit enhancements in brain-derived neurotrophic factor (BDNF) *in vivo* (Morales-García et al., 2017; de Almeida et al., 2019; Colaço et al., 2020; Morales-Garcia et al., 2020). At a behavioural level, single doses of ayahuasca have been demonstrated to occasion improvements in mood, empathy, creativity and satisfaction with life in (sub)clinical populations (Uthaug et al., 2018; Palhano-Fontes et al., 2019; Uthaug et al., 2021; van Oorsouw et al., 2022).

Given the neuroplastic effects of ayahuasca, a parsimonious explanation of sustained changes in behaviour and functional network dynamics seen following intake is that they are underpinned by changes to the anatomical organisation shaping cortical function. Attesting to this, prior work has demonstrated Santo Daime members can be distinguished from case-matched controls from a thinning of cortical midline structures such as the posterior cingulate cortex (PCC), a key hub of the default mode network and thickening of the isthmus of the corpus callosum (Bouso et al., 2015; Simonsson et al., 2022). However, it is still unknown how these group-wise univariate assessments may be reflective of 5-HT_2A_-mediated structural plasticity, let alone correspond to an individual participant’s anatomical organisation, which imposes strong constraints on whole-brain dynamics of functional networks (Bullmore and Sporns, 2012; Cabral et al., 2017; Hansen et al., 2022).

In recent years, tried-and-tested holistic approaches to structural neuroimaging such as morphometric similarity network (MSN) analysis which combines multiple morphological features from structural images, have been used to elucidate whole-brain anatomical networks for individual subjects (Seidlitz et al., 2018). By following the assumption that cortical regions which fire and wire together also share similar regional morphometric profiles (Goulas et al., 2017; Wei et al., 2018; Fulcher et al., 2019), MSNs have highlighted that cortical regions sharing a common cytoarchitecture are also likely to be anatomically connected. Since its conception, altered morphometric similarity (MS) has been shown to closely align with morphometric changes in a range of neuropsychiatric disorders sharing aberrant neuroadaptation as a hallmark as well as to predict individual differences in behaviour (Morgan et al., 2019; Seidlitz et al., 2020; Li et al., 2021; Wu et al., 2023). Edges (the pairwise relationship between two regions) comprising MSNs are closely associated with cortical fundamental properties, spanning gene expression, cytoarchitecture, and myeloarchitecture to evolutionary expansion (Seidlitz et al., 2018; Wei et al., 2018; Yang et al., 2021). Thus, MSNs provide an alternative neuroimaging phenotype useful for linking brain structural variation to neurogenetic markers of brain organisation.

Here, we sought to consolidate prior evidence of local structural differences following sustained psychedelic usage by using MSNs to explore global differences in anatomical network morphometry and tie them to neurogenetic markers of 5-HT_2A_-induced neuroplasticity. Leveraging the high signal-to-noise ratio afforded by 7T magnetic resonance imaging (MRI) in 24 Santo Daime members and a sample of matched controls, we tested the hypotheses that (i) repeat users would exhibit abnormalities in MSNs compared to controls (ii) MSN alterations would cluster within anatomical nodes pertinent to higher-order functional networks, and (iii) these differences would co-localise to transcriptional markers of 5-HT_2A_ expression.

## Methods

### Participants

The cohort comprised 24 volunteers (10 females, 55.2 [*SD*: 10.2] years) enrolled in a within-subject, fixed-order observational study conducted by Maastricht University as previously described (Mallaroni et al., 2022). Individuals were active members of the Dutch chapter of the church of Santo Daime who met the inclusion criteria comprising absence of ferromagnetic devices/implants (MRI contraindications), pregnancy, and use of (medicinal) substances in the past 24 h. Participants were highly experienced ayahuasca users with a mean (*SD*) membership duration of 14.2 (8.3) years, and a mean (*SD*) attendance of Santo Daime ceremonies of 563 (650) times. All participants gave written informed consent prior to scanning. The study was conducted according to the Declaration of Helsinki (1964) and amended in Fortaleza (Brazil, October 2013) and in accordance with the Medical Research Involving Human Subjects Act (WMO) and was approved by the Maastricht Academic Hospital and University’s Medical Ethics committee (NL70901.068.19/METC19.050).

Twenty-four healthy age (55.7, *SD* = 13) and sex (10 female) matched controls (age – *p* > 0.6266, CI [−1.73 – 2.81]) were randomly selected from the ‘Atlasing of the basal ganglia (ATAG)’ multimodal ultra-high resolution structural 7-Tesla MRI data repository (Forstmann et al., 2014). All participants had normal or corrected-to-normal vision, and none suffered from neurological, psychiatric, or somatic diseases.

### MRI acquisition

Whole-brain T_1_-weighted images (T1w) for the Santo Daime group were collected with a 7T Siemens Magnetom scanner (Siemens Medical, Erlangen, Germany) using 32 receiving-channel head array Nova coil (NOVA Medical Inc., Wilmington MA). The T1w images were acquired using a using magnetisation-prepared 2 rapid acquisition gradient-echo (MP2RAGE) sequence collecting 190 sagittal slices following parameters: repetition time (TR) = 4,500 ms, echo time (TE) = 2.39 ms, inversion times TI_1_/TI_2_ = 900/2750 ms, flip angle_1_ = 5°, flip angle_2_ = 3°, voxel size = 0.9 mm isotropic, bandwidth = 250 Hz/pixel.

T_1_-weighted images for the control group were acquired using a 7 T Siemens Magnetom MRI scanner using a 24 receiving-channel head array Nova coil (NOVA Medical Inc., Wilmington MA). An MP2RAGE acquisition collecting 240 sagittal slices with the parameters: TR = 5,000 ms, TE = 2.45 ms, inversion times TI_1_/TI_2_ = 900/2,750 ms, flip angle_1_ = 5°, flip angle_2_ = 3°, voxel size = 0.7 mm isotropic, bandwidth = 250 Hz/pixel.

MP2RAGE signal inhomogeneity was normalised by reconstructing “robust” T1w equivalents for all subjects as outlined by O'Brien et al. (2014). In sum, a normalised complexity ratio was extrapolated from T1w (GRE_TI1_) and PDw (GRE_TI2_) image volumes and applied to generate a uniform T1w image volume of minimal signal intensity variance (O'Brien et al., 2014). In addition outside of visual quality inspection, T1ws were assessed according to a set of quality control metrics: (i) coefficient of joint variation (CJV) assessing the presence of heavy head motion and large intensity nonuniformity artefacts (Ganzetti et al., 2016) (ii) contrast-to-noise ratio (CNR) an improvement of SNR to evaluate how separated the tissue distributions of GM and WM are (Magnotta et al., 2006), and (iii) the full-width half maximum (FWH) of the spatial distribution of the voxel intensity values, measuring the presence of image blur (Forman et al., 1995).

### Data preprocessing

Surface preprocessing of structural images was performed using the anatomical workflow of sMRIPrep 0.6.2 (as outlined here^1^) (Esteban et al., 2019). Briefly, T1w images were corrected for intensity nonuniformity with N4BiasFieldCorrection (ANTs) (Tustison et al., 2010) and skull-stripped with antsBrainExtraction.sh (ANTs). Skull-stripping was performed through OASIS template co-registration. Intensity-nonuniformity-corrected T1w volumes were then merged using reference subject T1w maps with mri_robust_template (FreeSurfer) (Fischl, 2012). Brain surfaces were then reconstructed and visually assessed using the subject’s T1w reference with recon-all (FreeSurfer) (Dale et al., 1999). Brain masks were estimated using a custom variation of a Mindboggle method (Klein et al., 2017) to reconcile ANTs-derived and FreeSurfer-derived segmentations of the cortical grey matter (GM). Brain tissues (cerebrospinal fluid [CSF], white matter [WM], and grey matter [GM]) were segmented from reference, brain extracted T1w images using FAST100 (FSL).

### Generation of MSN

Cortical surfaces were divided into 308 spatially contiguous nodes of approximately equal size (~5 cm^2^), derived from a subparcellation of the 68 cortical regions included within the Desikan-Killiany (DK) atlas (Desikan et al., 2006). This approach employs a backtracking algorithm to minimise the effect of inter-subject variability in parcel sizes defined by anatomical atlases (Romero-Garcia et al., 2012). We next transformed this parcellated DK atlas template to each participant’s native space using the inverse spherical normalisation parameters estimated during cortical surface reconstruction to avoid any further normalisation-induced heterogeneity. For each node, we extrapolated seven T1w morphometric features as per prior work (Seidlitz et al., 2018; King and Wood, 2020). Cortical thickness (CT), surface area (SA), mean (extrinsic) curvature (MC), Gaussian (intrinsic) curvature (GC), folding index (FI), curvature index (CI), and grey matter volume (GM). For each participant, morphometric feature vectors were z-scaled across regions to control for inter-feature variability. Pearson’s correlations were then performed for each pair of z-scored morphometric feature vectors, forming a 308 × 308 MSN per participant (Seidlitz et al., 2018).

### Case–control MSN analyses

Regional MS was calculated by summing weighted correlation coefficients between a given region and its correlations to all other regions. From this, the mean regional MS per condition can be derived by averaging across participants. To examine case–control differences, we fitted linear regression models (LRMs) to regional MS values and regressed out age, sex, and age x sex to further account for potential demographic differences. This model was fitted for each region, and the two-tailed *t*-statistic (contrast = ayahuasca – healthy controls [HCs]) was extracted. Significance was set at *p* < 0.05 with Benjamini–Hochberg false discovery rate (BH-FDR) for multiple comparisons across 308 regions. Furthermore, to contextualise macroscopic differences between groups, we referred them to two prior classifications of cortical areas (see Supplementary material for additional details): the Yeo 7 atlas of the cortex classified according to resting-state functional connectivity networks (Thomas Yeo et al., 2011) and the von Economo atlas of the cortex classified by cytoarchitectonic organisation (Scholtens et al., 2018). As a supplementary set of analyses, we also sought to assessed how changes in MS may influence the modular topology (Sporns and Betzel, 2016) of anatomical networks (their relative community structure and composition) using graph theory (see Supplementary material).

### Extraction and selection of regional gene expression values

To relate regional changes in MS to the cortical topography of gene expression for candidate receptors, we used cortical gene expression data from the publicly available Allen Human Brain Atlas (AHBA^2^). Regional gene expression levels for 20,000 + human genes were obtained microarray probes across hundreds of cortical loci in six postmortem brains from adult human donors with no history of psychiatric or neuropathological disorders (aged 24–57 years), as described in Hawrylycz et al. (2012). The AHBA dataset was preprocessed according to the steps outlined by Romero-Garcia et al. (2018) and mapped to our DK-308 parcellation. Since only two of the six AHBA brains included samples from the right hemisphere, we performed our transcriptomic analyses on 152 cortical regions in the left hemisphere in order to minimise lateralisation biases.

To reduce the dimensionality of our analysis, we defined apriori a set of 66 gene targets (152 regions x 66) encoding either (i) receptors/channels/transporters pertinent to ayahuasca’s binding profile (Ray, 2010) or (ii) an exploratory list of candidate neuroplasticity genes found to be differentially expressed following the acute administration of 5-HT_2A_ agonists as identified by de Vos et al. (2021). These targets not only included relevant primary receptors and transporters such as 5-HT_1A/2A/2C_, SIGMA-1, MAO_A/B_ but also neuroplasticity substrates such as NMDA/BDNF/cFOS/ARC/JUNB. For additional information pertaining exact gene targets, their respective studies, gene candidate selection criteria and their cortical distribution, see the Supplementary Tables S2, S3.

### Associating regional changes in MSN and transcriptomes

Following prior work (Morgan et al., 2019), we employed a partial-least-squares (PLS) regression approach to assess the relationship between left-hemispheric MSN differences (*t*-values) and transcriptional activity for our 66 gene targets. Gene expression values were used as predictor variables of regional changes in MS. PLS regression approaches are best suited in instances where the number of predictors exceeds the number of observations and when the predictors (genes) exhibit multicollinearity (Haenlein and Kaplan, 2004). The first component of the PLS (PLS1) was the linear combination of gene expression values that was most strongly correlated with regional changes in MS and provides an optimal low-dimensionality representation of the covariance of both variable sets. In order to assess the significance of the variance explained by PLS1, we permuted our response variables 10,000 times across extracted features as well as performed spin-permutation to assess the spatial relationship between our case–control MSN and PLS1 maps. We examined the relative contribution of each gene to PLS1 by using a bootstrapping procedure (random resampling and replacement of 152 regional values in 10,000 iterations) in which the variability of each gene’s occurrence in PLS1 was estimated, and the ratio of the weight of each gene to its bootstrap standard error is used to extrapolate a Z-score for each gene for ranking. Related genes for either positive, PLS1+, or negative, PLS1− were retained with a conservative confidence threshold of 99%.

### Quality control and replication analyses

Spin permutation testing was performed to mitigate potential confounding effects of spatial autocorrelations (Alexander-Bloch et al., 2018). Spatial maps were subject to 10,000 random spherical rotations at a vertex level to generate null models of spatial alignment. *P_spin_* value was computed as the proportion of null values of the intermodal Pearson correlation coefficient that were greater than the real values of the correlation coefficient. In order to assess the validity of our results we: (i) constructed MSNs using Spearman rank correlations (ii) incorporated total intracranial volume as a nuisance regressor in our LRMs of regional MS. For the latter, we extrapolated Jaccard Coefficient scores in order to compute the similarity between our main and replication results. Furthermore, we sought to assess prior findings of reduced CT in Santo Daime (Bouso et al., 2015). To do so, we fitted LRMs to regional CT values, while controlling for age, sex, age*sex and mean cross-hemispheric CT.

## Results

In order to assess structural differences associated to long-term ayahuasca use, we assessed MSNs in two imaging cohorts. Following quality control of images, we selected 24 Santo Daime members and matched them to 24 healthy controls. There were no significant (*p* > 0.05) between-group differences in the means of image quality, age, and sex (see Supplementary material).

### Repeated ayahuasca use is associated with altered MSN topography

**Figure 1:**
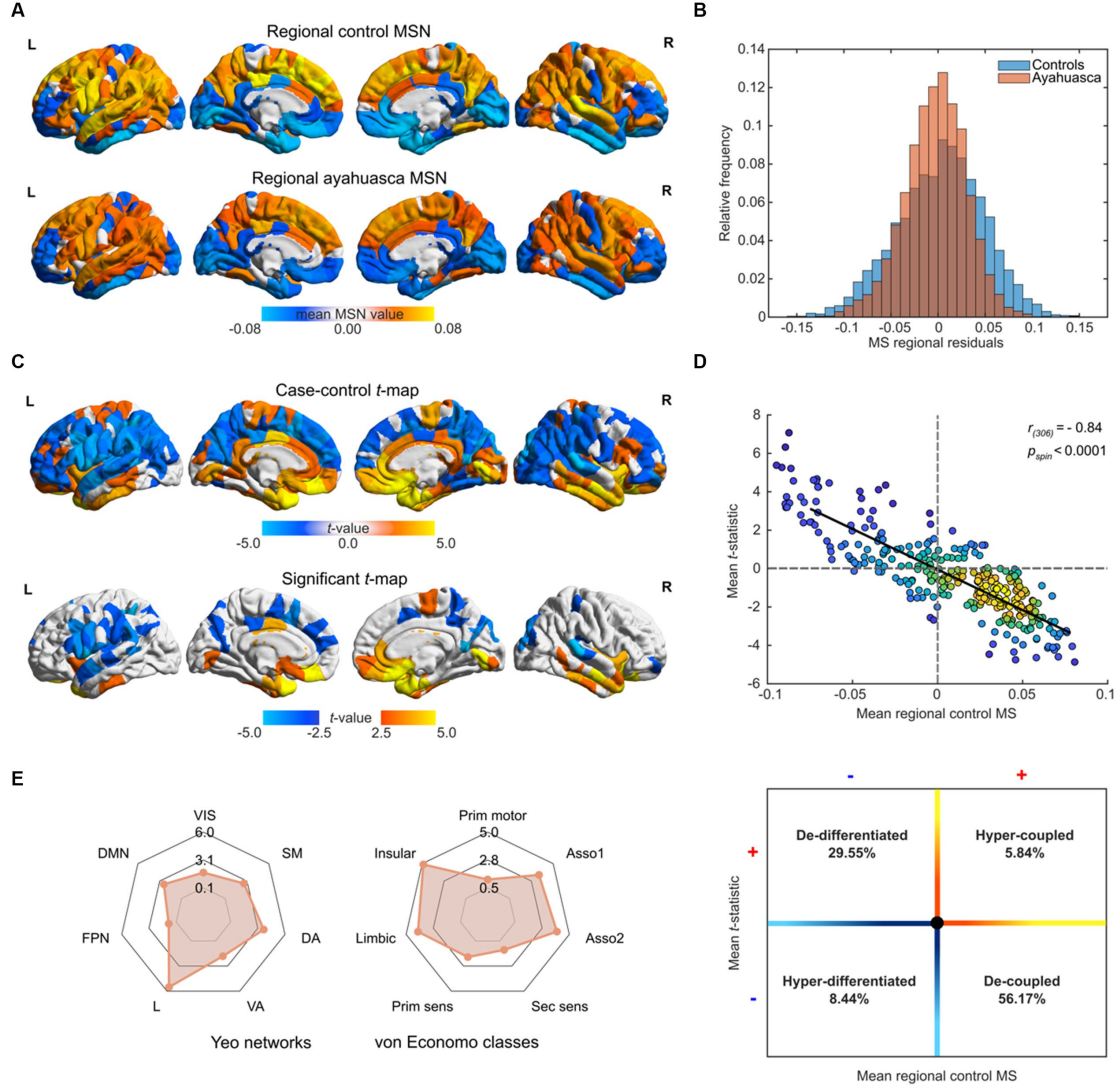
Morphometric similarity analyses of repeat ayahuasca usage. **(A)** Regional distribution of morphometric similarity (MS) in Santo Daime members and matched controls. **(B)** Case–control distributions of residual morphometric similarity, following regression of sex and age. **(C)** *t-*statistic and FDR flagged (*p* < 0.05) regions for differences in MS between groups (ayahuasca – controls). **(D)** Top – kernel density scatterplot of the mean regional MS scores of controls (*x*-axis) and the ayahuasca-control t-statistic (*y*-axis), bottom – schematic of functional implication of MS scatter plot value distribution. Lighter hues reflect higher value densities. **(E)** Case–control MS differences relative to Yeo functional and von Economo cytoarchitectural communities. Absolute *t*-statistics are displayed. Yeo abbreviations correspond to the following: VIS, visual network; DAN, dorsal attention network; SMN, somato-motor network; DA, dorsal attentional network; VA, ventral attention network; L, limbic network; FPN, fronto-parietal network; DMN, default mode network. Von Economo labels reflect the following: Prim motor, granular primary motor cortex; Asso1, granular association isocortex type I; Asso2, granular association isocortex type 2; Sec sens, secondary sensory cortex; Prim sens, primary sensory cortex; Limbic, limbic regions (allocortex including entorhinal, retrosplenial, presubicular and cingulate); Insula, insular cortex (containing granular, agranular and dysgranular regions). For all renders, local maximum values are displayed.

Overall, ayahuasca users exhibited diminished mean MS values compared to controls (*t* = 4.58, *p* < 0.0001), suggesting a predominant increase in anatomical differentiation. Within-group average summed weights of MSN values (308 regions) exhibited a normal distribution, balanced between regions of both high and low morphometric similarity (see Figure 1B). There was a significant difference between group distributions (*p* < 0.0001, two-sample Kolmogorov–Smirnoff test). Healthy control MSNs were found to show good spatial correspondence with 277 multimodal healthy control maps derived (*r*_(306)_ = 0.53, *p_spin_* < 0.0001) from prior work (Morgan et al., 2019) and constructed using additional DTI and T2 parameters at 3-Tesla. As demonstrated in Figure 1A, regions of high morphometric similarity largely loaded onto frontal and temporal cortical areas and high negative morphometric similarity onto occipital and motor cortices. MS value distributions were comparable to prior multimodal assessments in healthy individuals and reflect the notion that primary regions of the cortex are histologically differentiable from associative areas (Seidlitz et al., 2018; Vázquez-Rodríguez et al., 2019). MSN construction using a spearman rank approach yielded comparable regional residuals (*p_spin_* = <0.0001, *r*_(306)_ = 0.97).

We next assessed regional differences between ayahuasca users and controls by fitting a MLR on each region and produced two-sided, FDR-corrected mean *t*-statistic map. As shown in Figure 1C, repeat ayahuasca users exhibited decreased morphometric similarity in sensorimotor cortices (e.g., inferior frontal gyrus, precuneus, pre/post central gyrus) with increased morphometric similarity in primarily in midline, temporal and prefrontal structures (e.g., orbitofrontal, entorhinal, cingulate, anterior insular cortices). A reduction of regional MS in regular ayahuasca users group implies greater architectonic differentiation between specified areas and the rest of the cortex, which can be interpreted as reduced anatomical connectivity between less similar, more differentiated cortical areas, and conversely for regions expressing increased MS (see Supplementary Table S1 for regional values). The case–control *t*-map exhibited a strong negative spatial correlation with the mean control regional MS (Pearson’s *r_(306)_* = −0.84, *p*_spin_ < 0.0001, Figure 1D), indicating that more connected regions tend to show greater reductions in MS and vice versa. Positive regional *t*-values and negative mean MS representing regional architectonic de-differentiation in regular ayahuasca users in comparison to controls were found in 29.55% of examined regions, whereas 56.17% of regions held negative *t*-values and positive mean MS and reflecting regional architectonic differentiation (in other words, uncoupling) in ayahuasca users relative to controls. While changes in MSN composition were not mirrored by alterations to whole-brain structural modularity (*p* > 0.05), functional community affiliations were found to shift across modules (see Supplementary material).

To make our findings generalisable to other levels of brain organisation, namely, resting-state brain functional networks known to shift under 5-HT_2A_ agonists and cytoarchitectonic tissue classes, brain regions were also assigned to each of the Yeo 7 functional networks, as well as their corresponding von Economo cytoarchitectonic classes (Figure 1E). Here, ayahuasca users demonstrated decreased MS in Yeo SM, DA, and DMN networks (*p_FDR_* = 0.0165–0.0006) well as increased MS in the limbic (L) networks (*p* < 0.0001) For the von Economo classes, ayahuasca users had decreased MS in granular association isocortical classes types 1 and 2 (*p_FDR_* = 0.0010, 0.0003 respectively) and increased MS for limbic and insular classes (*p_FDR_*  = 0.0002, < 0.0001, respectively).

**Figure 2:**
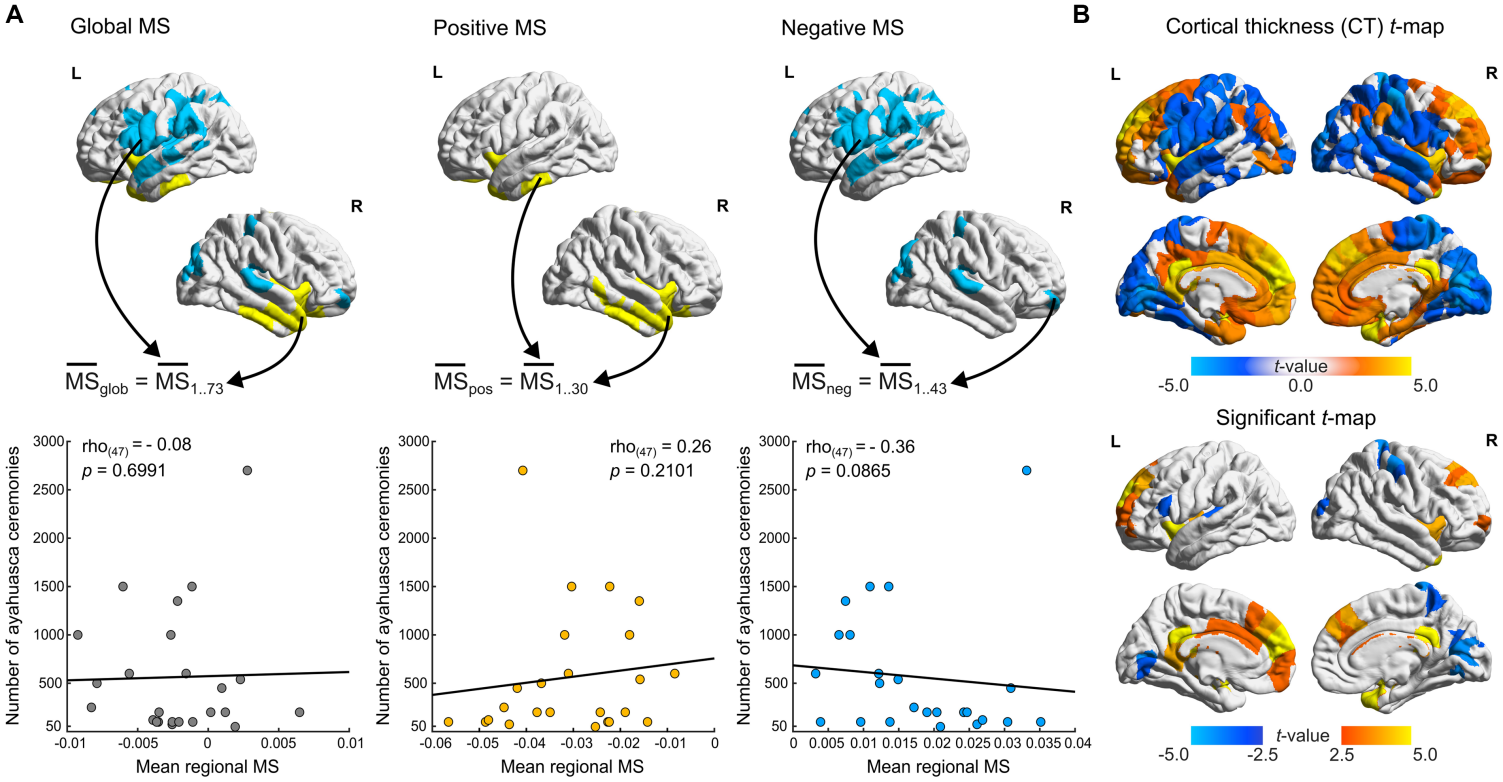
Cortical thickness and ayahuasca use frequency correlations. **(A)** Spearman correlations of ceremony attendance rates with MS scores. MS scores in FDR flagged regions are aggregated per contrast (positive negative and global, indicated by arrows) and averaged per participant. Scatter plots depict mean regional MS scores of Santo Daime members (*x*-axis) and corresponding ceremony attendance rates (*y*-axis). **(B)** *t-*statistic and FDR flagged (*p* < 0.05) regions for differences in CT between groups (ayahuasca – controls). For all renders, local maximum values are displayed.

Lastly, we sought to explore the relationship between ayahuasca use frequency and MS within our Santo Daime cohort. To do so, we employed two-tailed Spearman rank correlations to assess the relationship between ceremony attendance frequencies and mean FDR-flagged regional MS (significantly positive, negative and overall, see Figure 2A). A trend association was identified (max. Spearman’s *rho_(46)_* = −0.36, *p* = 0.0865).

### Replication analyses

A prominent nuisance covariate in volumetric analyses are variations in head size (Barnes et al., 2010), quantified by total intracranial volume (TIV). While no significant differences were found between groups, we validated the effect of TIV on our *t*-maps by including it as an additional nuisance regressor in our LRM. In this regard, FDR-flagged significant regions were largely congruent between methods (Jaccard = 90%, *t*-map *p_spin_* < 0.0001, *r* = 0.997, see Supplementary Table S1).

We also sought to reconcile the observed differences in our sample with prior findings of reduced CT in Santo Daime members (Bouso et al., 2015). As exemplified in Figure 2B and presented in Supplementary Table S1 and contrary to prior work, we identified opposing evidence of cortical thickening in midline structures and superior frontal regions (e.g., PCC, medial frontal cortex) as well as sparse cortical thinning in parietal and occipital regions (e.g., cuneus, postcentral). The resultant CT *t*-maps were found to be significantly associated with MSN *t*-maps (*p_spin_* < 0.0001, *r*_(306)_ = 0.39).

### Gene expression profiles mark alterations in MSN

**Figure 3:**
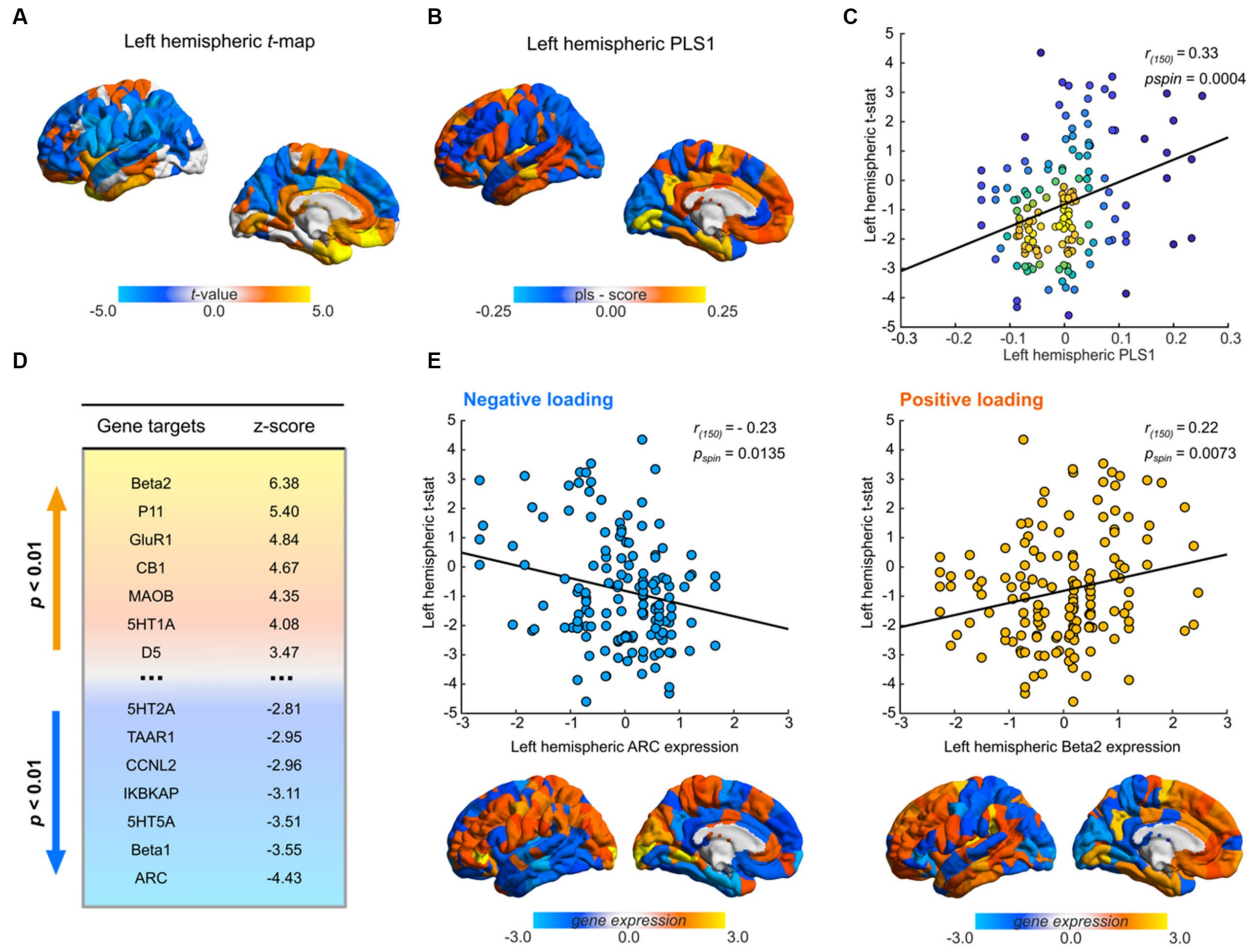
Transcriptional profiles associated with Santo Daime differences in morphometric similarity. **(A)** Cortical map of left hemispheric *t*-values used for PLS. **(B)** Regional loadings of PLS1 weights. **(C)** Kernel density scatterplot of the regional PLS1 scores of controls (*x*-axis) and regional ayahuasca-control left-hemispheric t-statistic (*y*-axis). Lighter hues reflect higher densities. **(D)** Significant PLS1 loadings following FDR correction. Gene targets reflect selected markers encoded by gene expression maps. Lighter hues representing positive loadings and vice versa. **(E)** Scatterplots of top gene target normalised gene expression values derived from the AHBA atlas in relation to regional differences in MS, paired with corresponding renders of their spatial distribution. For all renders, local maximum values are displayed.

To identify cortical transcriptional signatures of MSN differences under sustained ayahuasca use, we employed a PLS regression employing gene expression maps of 66 psychoplastogen targets (see Figures 3A,B). Following permutation testing (*p* = 0.0181), the first extracted component (PLS1) was retained and found to explain 11% of the case–control MSN *t*-map variance.

PLS1 gene expression weights exhibited a significant positive spatial correlation with MSN *t*-maps (Pearson’s *r_(150)_* = 0.33, *p_spin_* = 0.0004), signifying that genes which were positively weighted on PLS1 were overexpressed in regions demonstrating increased MS under ayahuasca relative to controls (Figure 3C), while genes which were negatively weighed in PLS1 were underexpressed in regions diminished MS. Closer examination (see Figure 3B) demonstrated that positive PLS1 gene expression weights strongly loaded onto prefrontal regions and conversely temporal regions for negative PLS1 gene expression weights.

As per Morgan et al. (2019), we then assessed the contribution of each target gene to PLS1 weights by employing a bootstrapping procedure to allocate relative z-scores. Overall, 18/66 genes were found to make significant contributions to PLS1 (*p* < 0.01, Figures 3D,E, see Supplementary Table S2 for a complete list). Among them, 11 genes had positive normalised PLS1 weights and were overexpressed in regions of high MS while 7 genes had negative normalised PLS1 weights and were underexpressed in regions of low MS.

## Discussion

We provide early evidence of altered structural network topography following sustained psychedelic usage. Partly consistent with our hypothesis, Santo Daime members exhibited a cortical patterning of significant increases in morphometric similarity in midline regions as well as significant reductions in associative sensorimotor cortices pertinent to functional and cytoarchitectural organisation. Beyond 5-HT_2A_ gene expression, case–control differences in morphometric similarity were more potently associated with receptors relevant to ayahuasca’s entourage effects on the human receptorome as well as a host of transcriptional factors and IEGs.

### Methodological considerations

By combining multiple structural features such as grey matter volume, cortical curvature or thickness, morphometric similarity approaches have been suggested to be a closer approximation of anatomical connectivity than univariate structural covariance approaches (Seidlitz et al., 2020). MSNs may therefore provide a clinically feasible proxy by which to assess structural connectomes in frequent circumstances where “ground-truth” axonal connectivity using DTI cannot be derived (King and Wood, 2020). While control MSNs were correspondent with prior multimodal work, observed differences in MSN topology observed in Santo Daime members are likely more indicative of cytoarchitectonic (de-)differentiation given the limited spatial specificity of surface macrostructural features.

### Shifts in morphometric similarity following repeated ayahuasca use

A hallmark of psychedelic-induced altered states of consciousness is their capacity to produce an acute loss of self-referential awareness, termed ego dissolution (Nour et al., 2016). Contrary to occasional users, Santo Daime members have been indicated to show a diminished susceptibility to ayahuasca’s effects on self-consciousness and perception (Ramaekers et al., 2023). The current findings of architectonic differentiation (denoted by decreased MS values) in cortices implicated in interoceptive and somatosensory functions (e.g., anterior insula, postcentral gyrus, precuneus) supporting both narrative and embodied self-consciousness (Blanke, 2012; Milliere, 2017; Chen et al., 2021; Skipper, 2022) may consequently underlie longer-term compensatory neuroadaptative changes. Current mechanistic frameworks of acute psychedelic effects propose that 5-HT_2A_ agonists disinhibit thalamocortical pathways serving to gate sensory influx, leading to increased activation of cortical somatosensory areas (Preller et al., 2019). Prior work has highlighted structural alterations as correlating with behavioural measures pertinent to selfhood (Bouso et al., 2015). Similarly, other ayahuasca studies have indicated changes in self-related measures after use (Bouso et al., 2012; Soler et al., 2016; Jiménez-Garrido et al., 2020; Kiraga et al., 2021).

Santo Daime members also exhibited architectonic de-differentiation (denoted by increased MS values) of regions relevant to emotional processing and experiential phenomena (limbic structures, eg. temporal poles) (Olson et al., 2007; Cristofori et al., 2016), executive control (prefrontal structures, eg. orbitofrontal cortex) (Friedman and Robbins, 2022) or serving as hubs for canonical resting-state networks (e.g., anterior cingulate – DMN) (Raichle, 2015). Transmodal nodes whose modular alliances swiftly change with task execution and hold extensive reciprocal projections to sensory and limbic modalities (Ghashghaei and Barbas, 2002), enable executive functioning and cognitive flexibility due to their role of mediating functional network reconfiguration (Braun et al., 2015; Medaglia et al., 2018; Finc et al., 2020). Prior evidence has demonstrated experienced ayahuasca users show a diminished susceptibility to drug-induced executive impairment relative to occasional users (Bouso et al., 2013) and exhibit distinct transmodal functional network connectivity acutely (Mallaroni et al., 2022). Furthermore, longitudinal assessments of Santo Daime have suggested members to exhibit better performance on measures of executive functioning and working memory (Bouso et al., 2012), while other cross-sectional studies indicate improved performance in verbal memory tasks compared to matched controls (Barbosa et al., 2016). Consequently, a structural de-differentiation of nodes with high modularity – that is, areas mediating long-distance connectivity between brain modules – may further underscore prior evidence of a functional tolerance to ayahuasca’s effects (Bouso et al., 2015). As a final point, it is also noteworthy that a reduction of topological centrality (or “hubness”) and local vulnerability of high-value nodes is considered to be a reliable transdiagnostic marker of neuropsychiatric disorders (Crossley et al., 2014; Hansen et al., 2022), given that repeated ayahuasca use is related to lower rates of psychopathology (Fábregas et al., 2010; Barbosa et al., 2012; Jiménez-Garrido et al., 2020).

We also sought to characterise how changes in MS may spatially relate to functional networks relevant to psychedelic effects. Differences in MS were diffuse across DMN, attentional networks (VA, DA) as well as primary sensorimotor (SM) and limbic (L) networks, correspondent with prior (sub-)acute functional imaging work (McCulloch et al., 2022). Furthermore, structural alterations coincided with specific cytoarchitectural classes, with differentiation being prominent within the isocortical areas comprising frontal and parietal lamination types while de-differentiation being present in the allocortex (limbic regions) and insular cortex (comprising granular, agranular and dysgranular lamination types). Whereas we had initially hypothesised morphometric differences would solely cluster in regions comprising transmodal functional networks with high 5-HT_2A_ receptor expression density, system-wide differences in functional connectivity in the form of a de-differentiation of hierarchical brain organisation are typically observed acutely under classical psychedelics (Girn et al., 2022; Timmermann et al., 2023). Considering that a ubiquitous principle of neuroadaptation is that sustained changes in functional connectivity are closely mirrored by structural adaptation, shifts in anatomical organisation may instead span a larger repertoire of networks.

It should be said that the full functional significance of the directionality of morphometric differences has yet to be established. While evidence of increased myelination or structural covariance between two cortical regions are typical precedents of structural plasticity (Cano et al., 2017; Kirby et al., 2022), the possibility remains that ‘less is also more’ in at least some cases: the phenomenon of synaptic pruning or hippocampal differentiation provides forceful examples (Low and Cheng, 2006; Diniz and Crestani, 2023). Consequently, it may instead be that particular anatomical regions are more labile/susceptible to neurogenesis as a result of differing microenvironmental properties (Bjornsson et al., 2015). Thus, an emphasis on regional differences (excluding demographic or methodological differences) may also help account for our findings of enhanced cortical thickness in cortical midline structures of Santo Daime members. In the present study, however, no clear link with use frequency was identified. Future longitudinal studies employing Santo Daime members at different stages of enrolment may provide a greater variance of use frequencies.

### Molecular profiles of altered morphometric similarity

In line with our hypothesis, 5-HT_2A_ gene expression was identified as a significant contributor to PLS1. Strikingly, factor loadings reflected a downregulation of 5-HT_2A_ receptor gene expression in sensorimotor cortices expressing greater morphometric differentiation. Similarly to currently approved psychiatric drugs, it is expected that the repeated use of psychedelic compounds affects the homeostasis of the 5-HT system via a sustained downregulation and desensitisation of 5-HT_2A_ receptors (Callaway et al., 1994; Romano et al., 2010; Raval et al., 2021). Tellingly, prior animal studies have confirmed a rapid downregulation of 5-HT_2A_ receptor expression in response to the repeated administration of LSD, concomitant to the onset of behavioural tolerance (Smith et al., 2014; Buchborn et al., 2018; Raval et al., 2021; de la Fuente Revenga et al., 2022).

As a botanical psychedelic exhibiting a complex polypharmacology, ayahuasca’s pharmacodynamics span a broad set of neuromodulatory systems. This is compounded by the inherent variability in the chemical composition of ayahuasca between communities, at times comprising additional minor psychedelic tryptamines such as for example, 5-hydroxy DMT (bufotenine) stemming from the use of *D. cabrerana* as a DMT source (Kaasik et al., 2021; Rodríguez et al., 2022). Here, we identified an extended combination of dysregulated serotonergic, aminergic, dopaminergic and cannabinoid receptor gene expression underlying morphometric differences in sustained ayahuasca users. It is generally understood that the pleiotropic effects of 5-HT_2A_ agonism are in part a consequence of downstream coupling with other Gq/11-coupled receptors (Inoue et al., 2019; Kim et al., 2020) identified herein (Lukasiewicz et al., 2010; Viñals et al., 2015; Moutkine et al., 2017). For example, *in vitro* assays have indicated acute stimulation of presynaptic 5-HT_2A_ receptors may regulate synaptic excitability by promoting the formation and release of the endocannabinoid 2-arachidonoylglycerol via an activation and subsequent downregulation of CB_1_ receptors (Parrish and Nichols, 2006; Best and Regehr, 2008). It is worthwhile noting alterations in peripheral primary endocannabinoids concentrations such as anandamide following acute ayahuasca intake have also been reported (dos Santos et al., 2022; Madrid-Gambin et al., 2022). It is also crucial to consider that indoleamines such as DMT are relatively nonselective 5-HT2 receptor agonists (Carbonaro and Gatch, 2016). Off-target partial agonism of receptors such as 5-HT_1A/2C_ or TAAR-1 are likely strong contributing factors to acute psychoactive effects of tryptamines (Canal et al., 2010; Pokorny et al., 2016; Shahar et al., 2022) and may consequently have neuroadaptive relevance. For example, Règue et al. (2019) have demonstrated 5-HT_2C_ overexpression may dysregulate BDNF and cytokine signalling. Furthermore, beyond MAO inhibition, b-carboline alkaloids such as harmine have also been found to have a non-specific binding profile with the exception of a modest affinity for a-adrenergic receptors (Buckholtz and Boggan, 1977; Drucker et al., 1990; Grella et al., 1998; Husbands et al., 2001; Grella et al., 2003).

By also exploring a subset of relevant genetic markers of neuroplasticity, the present analyses may help prioritise several pathways for future larger genetic association studies, comprising the totality of the AHBA transcriptome landscape. While the exact signalling cascades at play continue to be poorly defined, AMPA (glur1), TrkB, and mTOR and the subsequent promotion of IEGs such as ARC or JUNC, as seemingly necessary steps for psychoplastogen-induced neuronal growth (Ly et al., 2018, 2021; de Gregorio et al., 2021). Expression of plasticity-related genes required activation of both CaMKII and MAPK pathways (Desouza et al., 2021) and are closely regulated by transcriptional factors such as the S100A10 EF-hand protein (P11) or scaffolding proteins (IKAP), frequently implicated in neuropsychiatric disorders (George et al., 2013; Chottekalapanda et al., 2020). Furthermore, b-carboline alkaloids alone have been shown to assure neuroplasticity, cell survival and differentiation, BDNF expression, and inhibit both topisomerase and cyclin-dependent kinases (Fortunato et al., 2009; Sun et al., 2014; Morales-García et al., 2017; Pagano et al., 2017). In more recent years, both animal *in vivo* and human *in vitro* of models of 5-HT_2A_-mediated neuroplasticity have demonstrated differential expression of a sizeable number of genes (de la Fuente Revenga et al., 2021; Inserra et al., 2022; Kelley et al., 2022). Consequently, the present findings demand careful consideration given that the complex topographic interplay of employed genes and their regulatory mechanisms is far from fully understood nor can be modelled herein. Furthermore, with many of our epigenomic changes being isolated from rodent models, their transcriptional congruence with human models may vary. Cross-species pair approaches (animal to human) may have limited translatability depending on the gene in question given that sequence homology cannot be readily guaranteed (Naqvi et al., 2019).

### Limitations

While useful for establishing case–control differences in a target population, cross-sectional approaches such as those presented herein are not suited to derive direct causation. Aside from ayahuasca, it may be the case that other lifestyle factors inherent to Santo Daime, such as close social bonding, also drive morphometric differences (Taebi et al., 2020). Importantly, the reliability of any corollary associations is dependent on larger sample sizes and close sample matching. The present study employed external controls that could solely be matched on the basis of age and sex, and no other behavioural metrics relevant to morphometry such as verbal IQ or use frequency could be compared (Hyatt et al., 2020). Furthermore, practises pertaining to Santo Daime often regard ayahuasca as a medicinal sacrament, with members often originally enrolling with some form of psychopathology (Blainey, 2015) which may skew comparisons. While care was taken at a methodological level to ensure the reliability of our findings, particularly in relation to prior work by constraining our gene selection, differences in acquisition protocols between cohorts not flagged by our initial assessments may also in part contributed to morphometric differences. Lastly, while the AHBA atlas provides a complete mapping of relevant synaptic targets, otherwise inaccessible by PET atlases (e.g., BDNF), its postmortem gene expression maps are sparse (6 subjects) and likely closely covary with demographic variables such as age or sex (Arnatkeviciute et al., 2023).

To our knowledge, only one trial comprising 22 participants has previously sought to specifically address structural differences in Santo Daime congregants (Bouso et al., 2015). Brain-wide association studies of cortical features such as CT require thousands of individuals to generate robust phenotypes (Marek et al., 2022). Current global estimates of Santo Daime report between 4,000–7,875 active members (Blainey, 2015; Bastos et al., 2017), constraining attempts to gather suitable samples exempt from confounding psychopathology. Consequently, multi-centre trials pooling additional syncretic organisations such as União do Vegetal (UDV) or Barquinha (MacRae, 2004), as well as indigenous groups, could provide a fruitful venture for the study of repeat psychedelic use if approached in a culturally conscientious manner (Celidwen et al., 2023). Similarly, use of baseline structural data derived from prior studies of experienced ayahuasca users may also provide a suitable immediate compromise. Going forwards, paying closer attention to shifts in structural-functional coupling within holistic approaches informed by biophysical constraints, such as whole-brain models (Kringelbach et al., 2020), may provide predictive value for cohort-level differences in behaviour.

## Conclusion

Altogether, these findings provide initial evidence that repeat ayahuasca use is associated with changes in anatomical organisation underlying key functional networks. By using a pharmacologically informed approach, these results imply that downstream molecular mechanisms of psychedelics may ultimately connect to macroscale structural change in humans. Given the rare opportunity the ritualistic use of ayahuasca presents to study the persisting effects of psychedelics, future dedicated consortiums may prove useful in orchestrating assessments of neuroadaptive change.

## Data availability statement

The datasets presented in this study can be found in online repositories. The names of the repository/repositories and accession number(s) can be found in the article/Supplementary material.

## Ethics statement

The studies involving humans were approved by the Maastricht Academic Hospital and University’s Medical Ethics Committee. The studies were conducted in accordance with the local legislation and institutional requirements. The participants provided their written informed consent to participate in this study.

## Author contributions

PM collected the data, performed the analyses, and wrote the first version of the manuscript. NM, LK, and JTR collected the data and contributed to manuscript review. KO designed the Santo Daime study and contributed to manuscript review. JGR designed the Santo Daime study, contributed to manuscript review, and acquired funding. All authors contributed to the article and approved the submitted version.

## Footnotes

^1^ https://www.nipreps.org/smriprep/

^2^ http://www.brain-map.org
