# Supplementary material for: Cortical structural differences following repeated ayahuasca use hold molecular signatures
Source: Front Neurosci. 2023 Oct 5;17:1217079. doi: 10.3389/fnins.2023.1217079 (PMC10585114; doi:10.3389/fnins.2023.1217079)
Supplement: Supplementary file 2 [file Data_Sheet_1.docx]

Supplementary Materials for

**Cortical structural differences following repeated ayahuasca use hold molecular signatures**

^1^Pablo Mallaroni*****, ^1^Natasha L. Mason, ^1^Lilian Kloft, ^1^Johannes T. Reckweg, ^2^Kim van Oorsouw, ^1^Johannes G. Ramaekers*

**Author affiliations**

^1^Department of Neuropsychology and Psychopharmacology, Faculty of Psychology and Neuroscience, Maastricht University, P.O. Box 616, 6200 MD Maastricht, the Netherlands

^2^Department of Forensic Psychology, Faculty of Psychology and Neuroscience, Maastricht University, the Netherlands

**Corresponding authors’ emails***

[p.mallaroni@maastrichtuniversity.nl](mailto:p.mallaroni@maastrichtuniversity.nl), [j.ramaekers@maastrichtuniversity.nl](mailto:j.ramaekers@maastrichtuniversity.nl)

**Atlas allocation**

The augmented DK 308 parcellation was matched to two atlases defining functional organisation and cytoarchitectural tissue classes by Vértes et al. and Váša et al ^1,2^

*Yeo 7 atlas*

The 308 regions in our DK parcellation were matched to one of the seven cortical resting-state networks proposed by Yeo et al. ^3^ based on the greatest proportion of overlap of each region to each class. These networks included the visual (VIS), somatomotor (SM), dorsal attention (DA), ventral attention (VA), limbic (L), frontoparietal (FP) and the default mode network (DMN). The proportion of overlap was calculated in the *fsaverage* template volume per region as the number voxels within class, divided by the total number of voxels.

*von Economo atlas*

Each of the 308 regions delineated within the cortical parcellation framework underwent categorisation based on cytoarchitectural attributes following the classification of von Economo & Koskinas ^4^. The original classification system segregated the cortical regions into five distinct types, primarily based on their laminar structural characteristics, which broadly align with functional specialization in the cortex.

To elaborate, regions demonstrating limited differentiation in laminar structure, particularly exemplified by the primary motor cortex/precentral gyrus, were classified as structural type 1. Those regions typically recognized as association cortices were subcategorised into structural types 2 and 3, while secondary and primary sensory areas were designated as types 4 and 5, respectively.

The original classification framework lacked differentiation between the true six-layered isocortex and the mesocortex or allocortex, which exhibit substantial disparities in cytoarchitecture and developmental origins ^5^. To address this limitation, supplementary subtypes were generated: the limbic cortex, encompassing the entorhinal, retrosplenial, presubicular, and cingulate cortices, primarily representing allocortex; and the insular cortex, characterized by granular, agranular, and dysgranular regions, rendering it challenging to assign a single structural type. The assignment of structural types to cortical regions was carried out manually, guided by visual comparisons with von Economo & Koskinas's parcellation and informed by anatomical landmarks.

In sum, 7 cytoarchitectural were extracted: (1) granular cortex; primary motor/precentral gyrus, (2) granular association isocortex Type I, (3) granular association isocortex Type II, (4) secondary sensory cortex, (5) primary sensory cortex (6) limbic regions and (7) insular cortex (containing granular, agranular and dysgranular regions).

**Initial quality control**

**
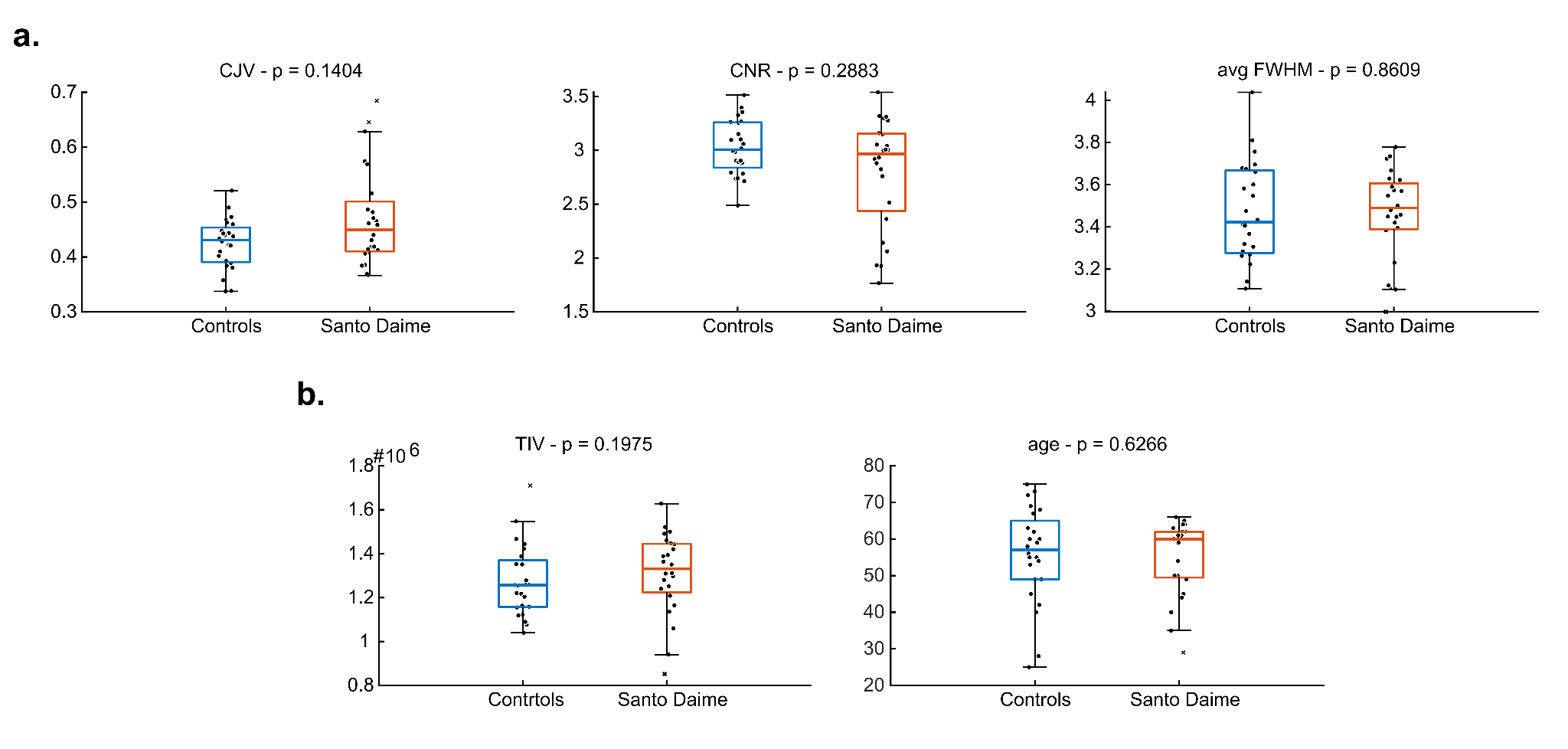
**

**Figure S1.** Quality control (QC) outcomes. **(a).** Boxplots of raw structural image noise metrics. *p*-values are derived from two-tail two-sample Wilcoxon rank sum testing. Note raw image QC measures can exhibit distinct behaviour as a result of heavy intensity background artefacts produced by MP2RAGE sequences prior to correction. **(b).** Boxplots of sample demographic differences. Independent sample two-tailed *t*-tests did not reveal significant differences in total intracranial volume nor age. Each group was sex matched (females = 10).

**Primary analysis replication accounting for total intracranial volume (TIV)**


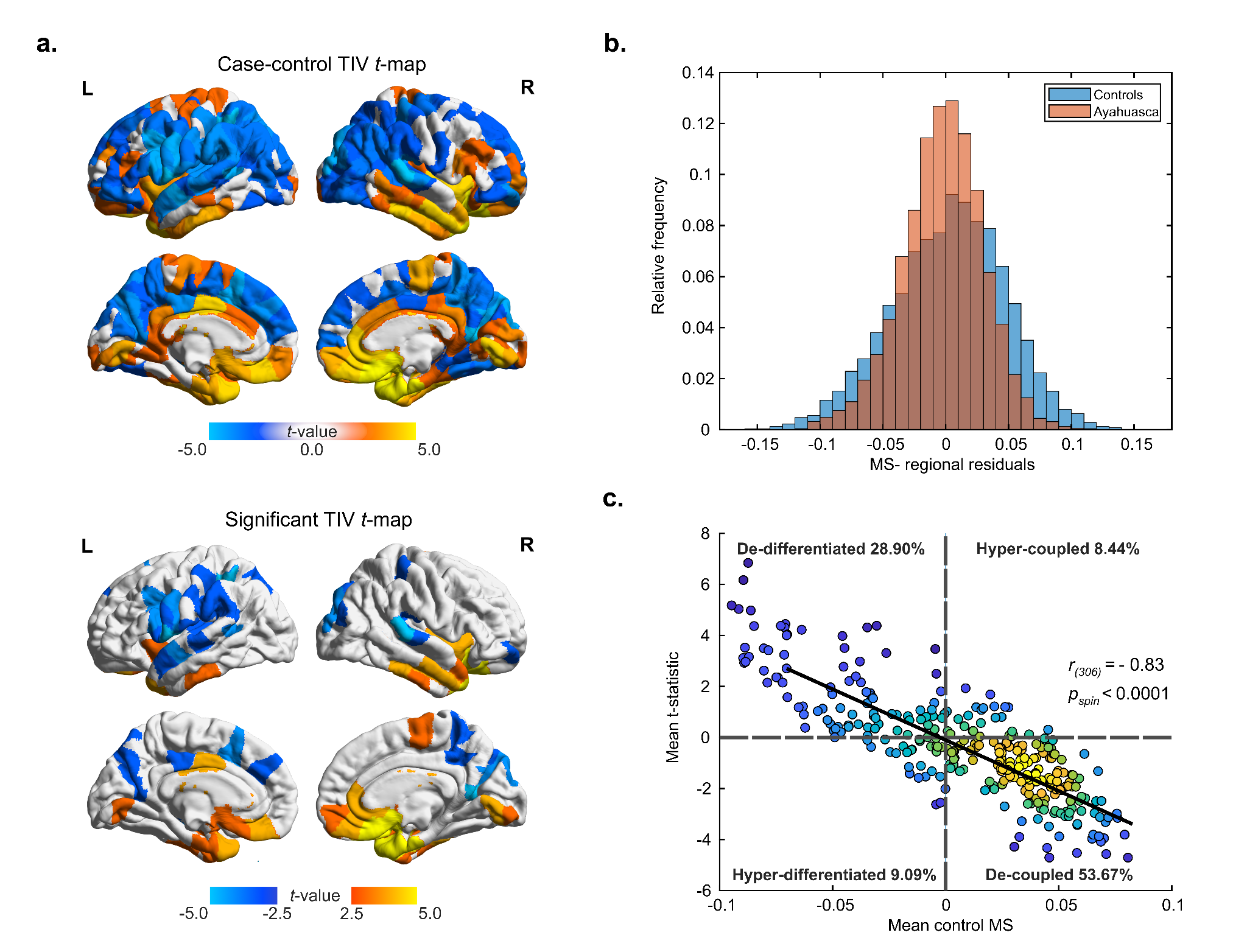


**Figure S2.** MSN analyses employing TIV as a nuisance regressor. **(a).** *t*-statistic and FDR-flagged (*p* < 0.05) regions for differences in MS between groups (ayahuasca – controls) following multilinear regression of age, sex, age*sex and TIV. **(b).**  Case-control distributions of MS regional residuals, following regression of age, sex, age*sex and TIV. There was a significant difference between group distributions (*p* <0.0001, two-sample Kolmogorov–Smirnoff test). **(c).** Kernel density scatterplot of the mean regional MS scores of controls (x-axis) and the ayahuasca-control t-statistic (y-axis). As found in our primary analyses, sustained use of ayahuasca was associated with an equivalent pattern of regional decoupling (high morphometric similarity shifting to low) and de-differentiation (low morphometric similarity shifting to high) and. Mean control MS maps were similarly correlated (Pearson) to correspondent *t-*maps.

**Cortical variation in target gene expression**

**
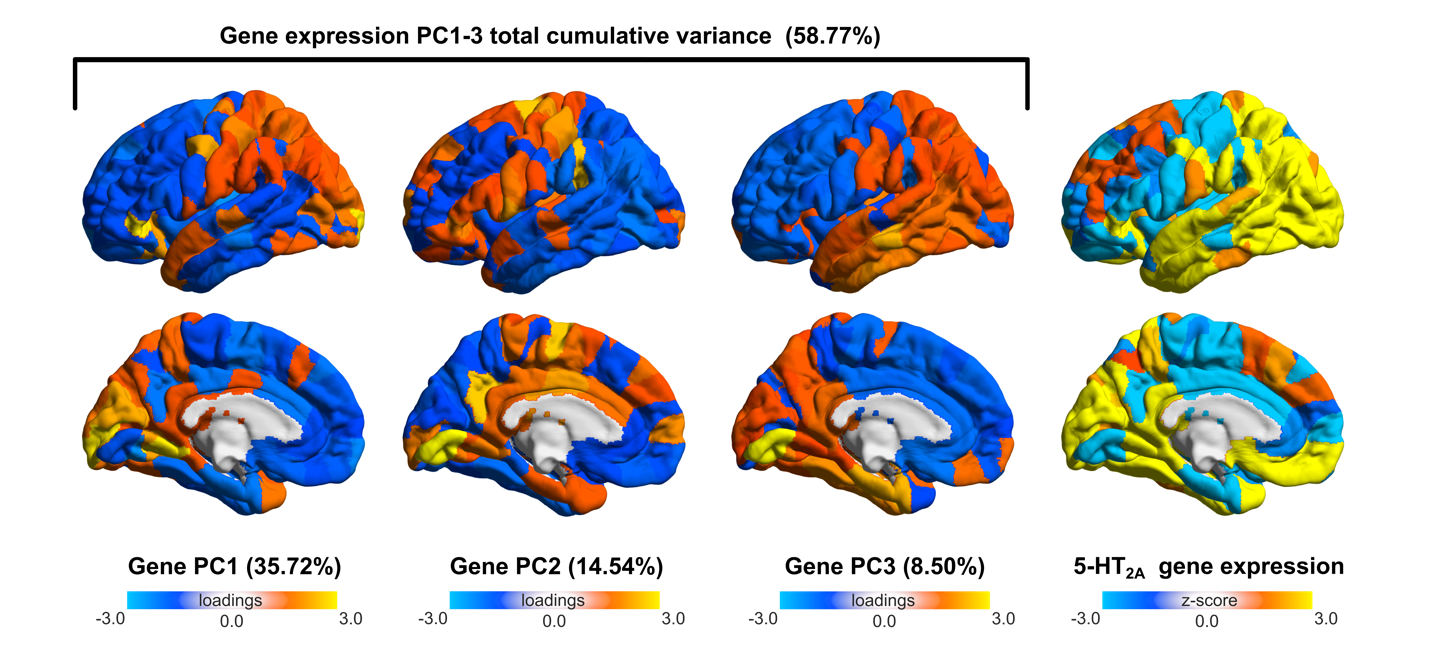
**

**Figure S3.** Brain renders of left hemispheric cortical variation of selected target genes (n = 66), including primary 5-HT_2A_ measure. Loadings of the first three principal components (PC1-3) of cortical variation in gene expression from the 66 target genes isolated from the Allen Human Brain Atlas transcriptomic database (see tables S2, S3) are provided alongside normalised expression values for 5-HT_2A_ gene expression.

**Network MSN-transcriptional phenotypes**

**
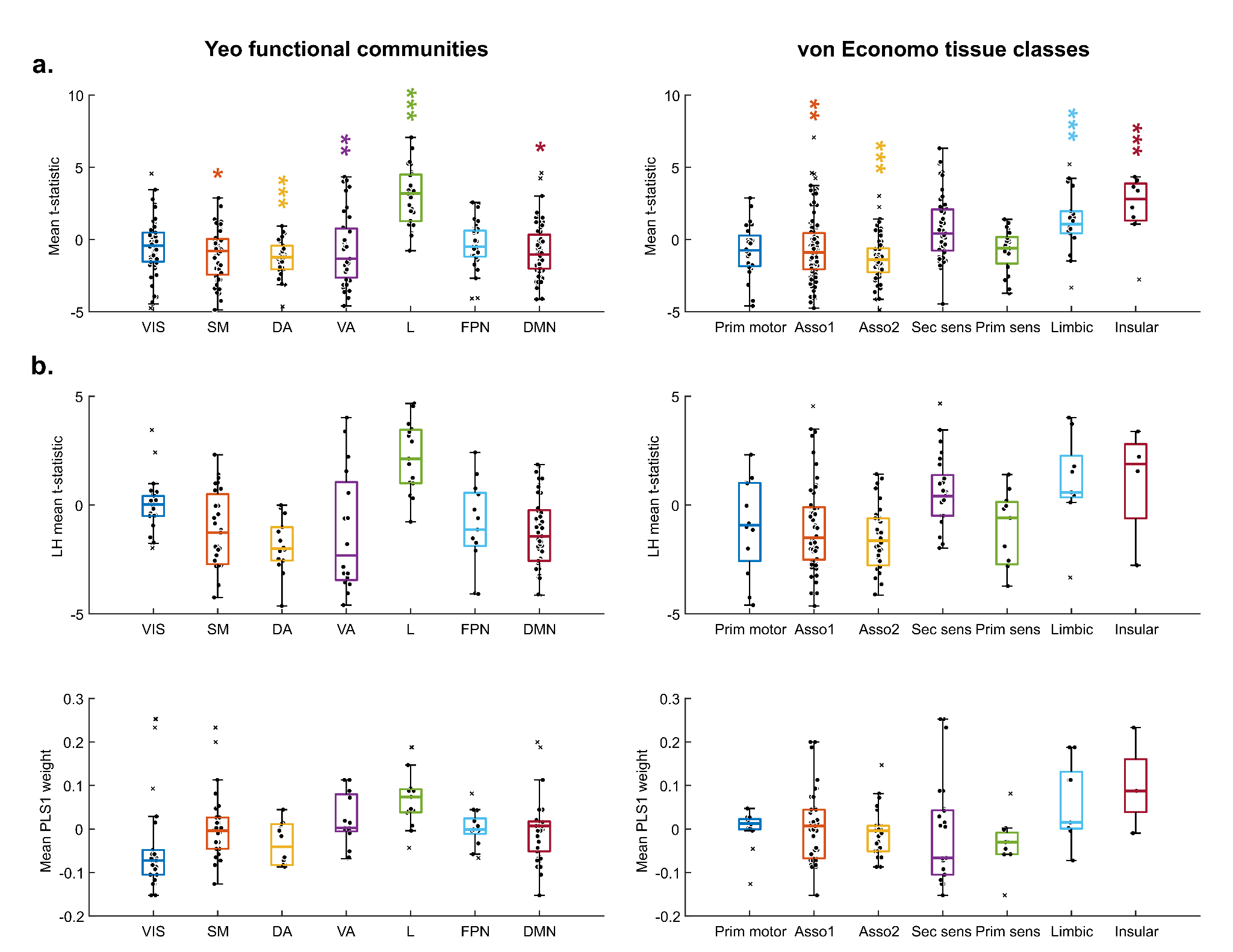
**

**Figure S4.** Yeo networks and von Economo classes. **(a).** Boxplots showing whole-brain mean *t*-statistic per Yeo functional network and von Economo tissue classes. Significant MLR group differences (reported in the manuscript figure 1) following FDR correction are indicated according to *p*> 0.05 *, *p*>0.01**, *p*>0.001***. **(b).** Boxplots showing left-hemispheric mean t-statistic per Yeo functional network and von Economo tissue classes in relation to PLS1 loadings. From inspection, it can be noted the differences in PLS1 scores between the Yeo and von Economo categorisation only closely follow MS *t*-statistics (or differences) for a select number of categories (eg. Limbic, Insular). For each atlas, one-way Kruskal-Wallis tests demonstrated variation in PLS (Yeo. *p*<0.0001, von Economo. *p* = 0.0309) and t-scores (Yeo. *p*<0.0001, von Economo. *p*<0.0001), reflecting a significant distribution of scores across categories. VIS visual network, DA dorsal attention network, SM somatomotor network, VA ventral attention network, L limbic network, DMN default mode network, FPN frontoparietal network.

**Adaptations in modular MSN structure**

*Method and rationale*

Prior sub(acute) resting-state functional connectivity studies using serotonergic psychedelics have highlighted a small-word functional architecture, characterised by greater global brain network integration across a range of graph theoretical measures ^6-8^. Changes to functional brain modularity have been previously highlighted as relevant markers of intervention-related neuroplasticity and prove to be a useful marker of network segregation/integration ^9,10^. Importantly, structural network modules develop in a similar manner as functional brain networks and in turn, define global cortical connectivity^11^. Given prior evidence of subacute shifts in modularity following single administrations of psychedelics and enhanced structural plasticity ^8,12^, we therefore sought to assess whether changes to the modular architecture of functional connectivity may also extend to a structural level of organisation.

We first quantified global modularity based on the degree of within-module connections compared to between-network connections ^13^. Higher modularity reflects a greater number of within-module connections and fewer connections between modules. A highly modular brain can be interpreted as a brain that contains highly specialized brain networks with less integration between networks. Subject-level network modules and scores were identified using consensus community detection in which an unsupervised Louvain community detection algorithm is employed to detect module arrangements ^10,14^. Since modularity is reliant on the network’s total MS score, we normalized each modularity score by dividing it by the mean of the corresponding null distribution calculated on a set of 100 randomly permuted original networks ^15^. This approach also serves to account for the stochastic nature of unsupervised approaches such as those presented herein.

In parallel, we assessed how cytoarchitectural and functional affiliations of identified modules may change because as a function of altered MSN topology. Generated subject-level module arrangements were integrated to generate a co-classification matrix, with entry (i, j) represents the frequency of region i and j being clustered into the same community. Louvain community detection was next applied to this co-classification matrix to generate a group-level consensus of community membership, with the resolution parameter *γ* set as the default parameter (1). This approach yielded three module clusters per group (Figure S5b). Lastly, we extrapolated group-level changes to the spatial overlap of identified clusters with canonical Yeo resting-state networks and cytoarchitectural von Economo tissue classes by extrapolating Dice coefficient scores per module-atlas pair.

*Results*

As demonstrated in Figure S5a, structural MSNs exhibited highly modular characteristics. Globally, two-tailed Wilcoxon sign rank testing did not reveal significant differences in modularity attributable to sustained ayahuasca usage (*p* = 0.9918), thereby implying that the global degree of MS network specialisation was preserved relative to controls. At a local level however, spatial cluster (Figure S5b) arrangements identified under ayahuasca exhibited distinct shifts relative to controls. Modules exhibited a reallocation of spatial affiliations, with communities such as for example community 1, otherwise closely overlapping with the DMN and VIS networks instead now becoming less spatially defined and comprising alternative systems such as L (Figure S5c). All in all, shifts in a community spatial overlap may further reflect a structural reconfiguration of regional MSN, in that module arrangements no longer follow otherwise “normative” functional or cytoarchitectural axes of organisation.

**
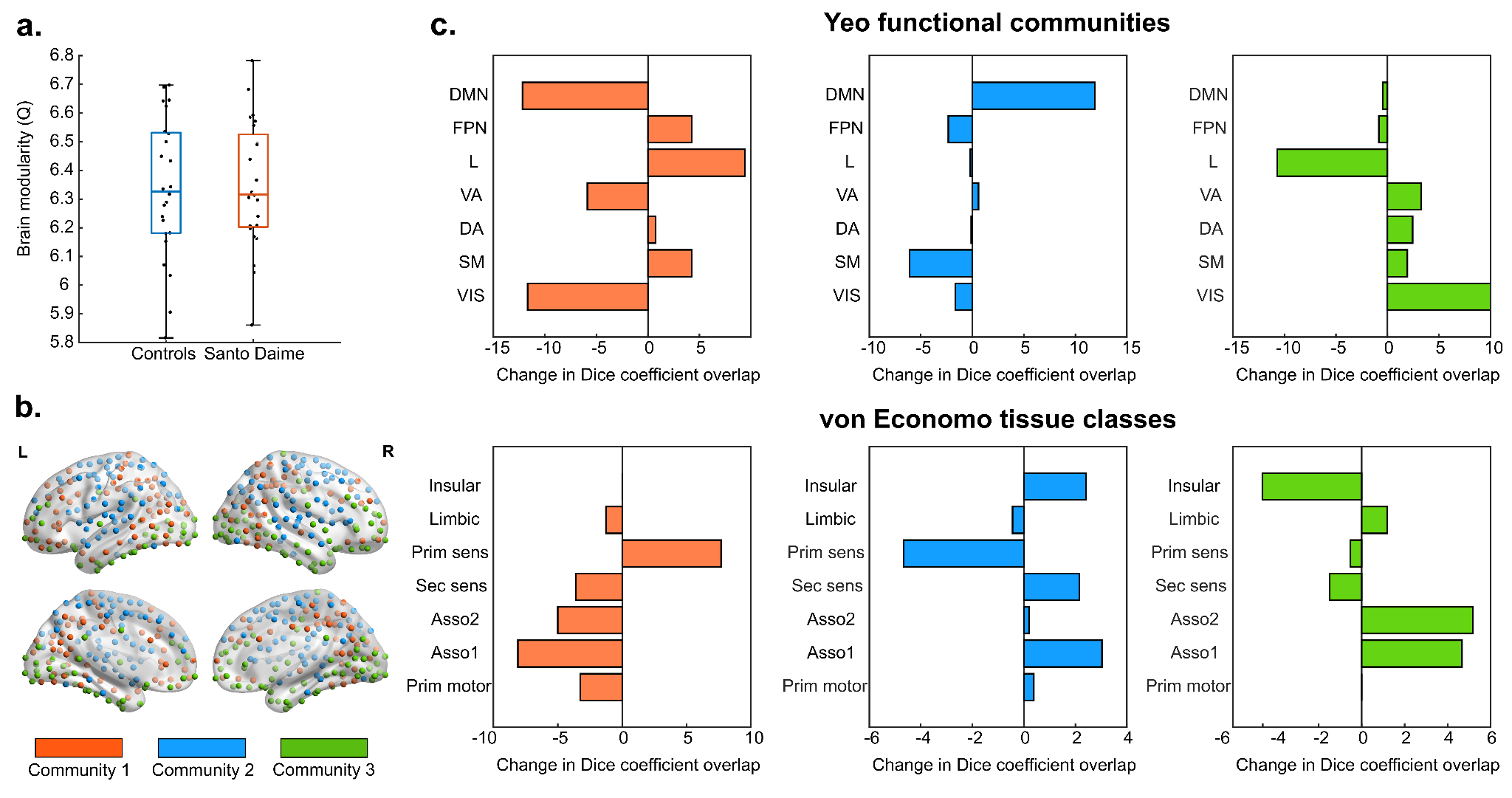
**

**Figure S5.** Changes to modular organisation of MSNs **(a).** Boxplots showing whole-brain structural modularity (Q) scores per group. No significant differences were identified. **(b)**. Modular structure of Santo Daime MSNs. Three group-level communities were identified using a Louvain community detection approach. Contributing nodes are colour-coded according to their respective community. **(c)**. Shifts in the spatial overlap of MSN community affiliation. Panels represent lambdas of Dice coefficient overlap scores between groups (Santo Daime – controls), derived for each Yeo and von Economo class. Panels are colour coded according to the module in questions (S5b)

**References**

1 Vértes, P. E. *et al.* Gene transcription profiles associated with inter-modular hubs and connection distance in human functional magnetic resonance imaging networks. *Philosophical Transactions of the Royal Society B: Biological Sciences* **371**, 20150362 (2016). <https://doi.org:doi:10.1098/rstb.2015.0362>

2 Váša, F. *et al.* Adolescent Tuning of Association Cortex in Human Structural Brain Networks. *Cerebral Cortex* **28**, 281-294 (2017). <https://doi.org:10.1093/cercor/bhx249>

3 Yeo, B. T. *et al.* The organization of the human cerebral cortex estimated by intrinsic functional connectivity. *J Neurophysiol* **106**, 1125-1165 (2011). <https://doi.org:10.1152/jn.00338.2011>

4 Scholtens, L. H., de Reus, M. A., de Lange, S. C., Schmidt, R. & van den Heuvel, M. P. An mri von economo–koskinas atlas. *NeuroImage* **170**, 249-256 (2018).

5 Mai, J. K. & Paxinos, G. *The human nervous system*. (Academic press, 2011).

6 Singleton, S. P. *et al.* Receptor-informed network control theory links LSD and psilocybin to a flattening of the brain’s control energy landscape. *Nature Communications* **13**, 5812 (2022). <https://doi.org:10.1038/s41467-022-33578-1>

7 Luppi, A. I., Carhart-Harris, R. L., Roseman, L., Pappas, I., Menon, D. K. & Stamatakis, E. A. LSD alters dynamic integration and segregation in the human brain. *Neuroimage* **227**, 117653 (2021). <https://doi.org:10.1016/j.neuroimage.2020.117653>

8 Daws, R. E. *et al.* Increased global integration in the brain after psilocybin therapy for depression. *Nature Medicine* **28**, 844-851 (2022). <https://doi.org:10.1038/s41591-022-01744-z>

9 Gallen, C. L. & D'Esposito, M. Brain Modularity: A Biomarker of Intervention-related Plasticity. *Trends Cogn Sci* **23**, 293-304 (2019). <https://doi.org:10.1016/j.tics.2019.01.014>

10 Sporns, O. & Betzel, R. F. Modular Brain Networks. *Annual Review of Psychology* **67**, 613-640 (2016). <https://doi.org:10.1146/annurev-psych-122414-033634>

11 Baum, G. L. *et al.* Modular Segregation of Structural Brain Networks Supports the Development of Executive Function in Youth. *Current Biology* **27**, 1561-1572.e1568 (2017). <https://doi.org:https://doi.org/10.1016/j.cub.2017.04.051>

12 Calder, A. E. & Hasler, G. Towards an understanding of psychedelic-induced neuroplasticity. *Neuropsychopharmacology* **48**, 104-112 (2023). <https://doi.org:10.1038/s41386-022-01389-z>

13 Newman, M. E. J. & Girvan, M. Finding and evaluating community structure in networks. *Physical Review E* **69**, 026113 (2004). <https://doi.org:10.1103/PhysRevE.69.026113>

14 Fortunato, S. Community detection in graphs. *Physics reports* **486**, 75-174 (2010).

15 Maslov, S. & Sneppen, K. Specificity and stability in topology of protein networks. *Science* **296**, 910-913 (2002). <https://doi.org:10.1126/science.1065103>
